# Efficacy of Entomopathogenic Fungi as Prevention against Early Life Stages of the Red Palm Weevil, *Rhynchophorus ferrugineus* (Coleoptera: Curculionidae) in Laboratory and Greenhouse Trials

**DOI:** 10.3390/insects14120918

**Published:** 2023-11-30

**Authors:** Dana Ment, Noa Levy, Arnon Allouche, Michael Davidovitz, Gal Yaacobi

**Affiliations:** 1 Department of Plant Pathology and Weed Research, Plant Protection Institute, Agricultural Research Organization (ARO), The Volcani Center, HaMaccabim Road 68, Rishon LeZion 7528809, Israel; noalevy8923@gmail.com; 2 BioBee Ltd., Sde Eliyahu 1081000, Israel; arnon_a@biobee.com; 3 Department of Entomology, Plant Protection Institute, Agricultural Research Organization (ARO), The Volcani Center, HaMaccabim Road 68, Rishon LeZion 7528809, Israel; michaeld@volcani.agri.gov.il

**Keywords:** microbial control, *Metarhizium*, *Beauveria*, biopesticide, red palm weevil, prevention

## Abstract

**Simple Summary:**

Public demand for the reduction, or even total elimination, of chemical residues in agricultural products, coupled with increased concerns about the detrimental environmental effects of chemical or synthetic pesticides, facilitated the need to develop alternative pest control means in agriculture. Microbial control agents (MCAs) are considered an attractive alternative to chemical insecticides due to ecological safety with no residues, wide host range, and competitive prices. Here, we evaluated several insect-killing fungi as a preventative control measure against the destructive palm pest, the red palm weevil (RPW), in lab experiments and in the greenhouse. The fungi were applied on healthy Washingtonia palms as a protective mean prior to being challenged with the RPW. The results demonstrated that treated palms were significantly less colonized by the RPW, and the fungi prevented the establishment of this severe pest. This study is the first stage in our bigger research endeavor for protecting date palm plantations with EPF.

**Abstract:**

The red palm weevil (RPW) *Rhynchophorus ferrugineus* is a highly destructive invasive pest for palms whose management is mainly by application of synthetic pesticides. As a key pest of date palm plantations, it is necessary to integrate environmentally safe measures for its management. Entomopathogenic fungi (EPF) have been primarily studied as a preventative control measure due to the horizontal transfer of conidia within the RPW population. We previously demonstrated the horizontal transmission of fungal conidia from an egg-laying surface to the female weevil and then to the eggs and larvae. Based on that strategy, this study aimed to evaluate the virulence of commercial EPF products and laboratory EPF preparations to RPW females and their progeny, and their ability to protect palms against infestation. As such, it serves as a screening platform for field experiments. Mortality rates of females and eggs depended on the applied treatment formulation and fungal strain. Velifer^®^, a *Beauveria bassiana* product, and *Metarhizium brunneum* (Mb7) resulted in 60–88% female mortality. Mb7—as a conidial suspension or powder—resulted in 18–21% egg-hatching rates, approximately 3 times less than in the non-treated control. Treating palms with Mb7 suspension or dry formulation significantly inhibits infestation signs and results in protection. These results lay the foundation for investigating the protective rate of EPF products against RPW in date plantations.

## 1. Introduction

The red palm weevil (RPW) *Rhynchophorus ferrugineus* (Olivier) (Coleoptera: Curculionidae) is an invasive pest in the Mediterranean basin and the Middle East [1]. It is a severe pest of palms, particularly the genus *Phoenix*. As in several other countries of the Mediterranean basin, RPW is a significant problem in Israel [2,3]. The canary palm, *Phoenix canariensis*, is the most susceptible palm specie to RPW in the Mediterranean, but it also poses a significant threat to date palms (*Phoenix dactylifera*) (Red Palm Weevil, 2020). To date, attempts to limit the damage caused by RPW have relied mainly on the application of synthetic pesticides: neonicotinoids as preventative treatments and pyrethroids as reactive measures [4,5,6]. Date palm infestation rates as detected by seismic sensors can range between 0.5 and 9.5% depending on management practices [7]. As infestation rates rise, the risk of palm collapse rises as well.

Biological control of RPW is limited because this weevil has a negligible activity of natural enemies in its native habitat [8,9]. Efforts to develop biological control management against RPW have focused on microbial control agents: entomopathogenic nematodes, e.g., *Steinernema carpocapsae* and *Heterorhabditis bacteriophora* [5,10,11,12,13], and various genera and species of entomopathogenic fungi (EPF) [9,14,15,16,17,18,19,20]. The use of EPF as microbial control agent against RPW has been suggested in several studies (reviewed by 9). There are a variety of strategies for applying EPF products against RPW, including both inundative and inoculative applications, involving direct application of the EPF products to palm trees [21] or attract-and-infect traps [16,22]. The common principle underlying these strategies is the attraction of adult RPW to a specific site where the EPF is applied. The adult weevils that come into contact with the EPF conidia then disseminate those conidia among the RPW population. Vectoring of the conidia can result in infection of RPW progeny via egg contamination during the oviposition process, as in Matveev et al. [23], who demonstrated the mechanical transfer of conidia between females and progeny. In that study, transmission resulted in inoculation of the egg-laying hole, reducing egg hatch and larval survival. In studies involving field trials, EPF was applied on the palm at the typical RPW infestation sites—the crowns of canary palms [21] and the trunks of date palms [17]. Other strategies for EPF application have been proposed, such as auto-contamination traps [24] and attract-and-infect traps [16,22]. These strategies rely on the passive acquisition of conidia and their mechanical transmission among the adult RPW population. 

The larval stages of RPW are cryptic, and they complete their life cycle inside the palm tissue. Mechanical transmission of conidia that is effective enough to infect the larvae and thus reduce palm infestation rates is an interesting strategy. The oviposition behavior of the female weevil as an opportunity for conidial transmission toward the laying hole was recently examined by Matveev et al. [23], demonstrating the mechanism governing this conidia transmission. The objectives of this study were to evaluate (1) the virulence of commercial EPF products and laboratory preparations of EPF by assessing their effect on RPW female survival, egg hatching, and larval survival in a microcosm designed to enable transmission of conidia into the egg-laying hole; and (2) the efficacy of the most promising EPF from objective 1 in protecting palms from RPW infestation under greenhouse conditions. This type of screening will enable further evaluation of the most promising EPF products in field trials that are now being conducted in Israeli date palm plantations. 

## 2. Materials and Methods

### 2.1. Entomopathogenic Fungi

*Metarhizium brunneum* 7 (Mb7) was routinely grown on Sabouraud dextrose agar (SDA; Difco) for 2 weeks at 28 °C. Mb7 was produced by solid-state fermentation on rice to prepare conidial powder. Conidia were separated from the rice by sieving through a 12-mesh sieve, and harvested conidia were stored at 4 °C in a sealed plastic box. For the Mb7 suspension, conidia were harvested by scraping the agar and putting the scrapings into glass tubes with sterile distilled water containing 0.01% (*w*/*v*) Triton X-100. The suspensions were vortexed and filtered through Miracloth (Calbiochem, La Jolla, CA, USA), and spore concentrations were determined with a hemocytometer. Suspensions were adjusted to the required conidial concentrations in 0.01% Triton X-100, and the percentage of viable conidia was determined on SDA before each bioassay. Only conidial suspensions with at least 95% germination were used for bioassays. 

The evaluated commercial products were two *Beauveria bassiana* strains (Table 1). To date, only Botanigard^®^ (LAM International Corporation, Butte, MO, USA) is registered and commercially available in Israel. Velifer^®^ (BASF, Gqeberha, South Africa) is under evaluation for the registration process. Both *B. bassiana-based* products are evaluated for commercial use against the RPW; therefore, they are a necessity for the lab assay for virulence evaluation. Descriptions of the fungal species and strains used in the bioassays are presented in Table 1. The above-described viability test was also performed for all of the products and strains examined in this study, and only EPF with at least 95% germination were used.

### 2.2. Insects

The RPW adults used in the bioassays were provided by the Plant Protection and Inspection Services of the Israeli Ministry of Agriculture (PPIS). Briefly, insects were trapped by Picusan^®^ traps (SANSAN PRODESING SL, Valencia, Spain) containing pheromone–kairomone lures (4-methyl-5-nonanol and 4-methyl-5-nonanone, ethyl acetate and sugar molasses), supplied by Biobee Ltd., Sde Eliyahu, in northwestern Israel and along the Israeli coastline. The trapped insects were sexed for seven days in a male/female ratio of 2:3. Adult sexing and maintenance were performed in groups of up to 50 adults in plastic boxes (20 cm wide × 40 cm long × 20 cm high). Each box had four 10 cm diameter mesh-covered holes for ventilation. The insects were fed on fresh sugarcane. Boxes were kept in environmental chambers at 25 °C and 70% relative humidity (RH) with a 10 h:14 h dark/light regime. Females that were not laying eggs were discarded from the experiments as it suggests that their processing was not performed well or they are infertile.

### 2.3. Laboratory Bioassay

**Figure 1:**
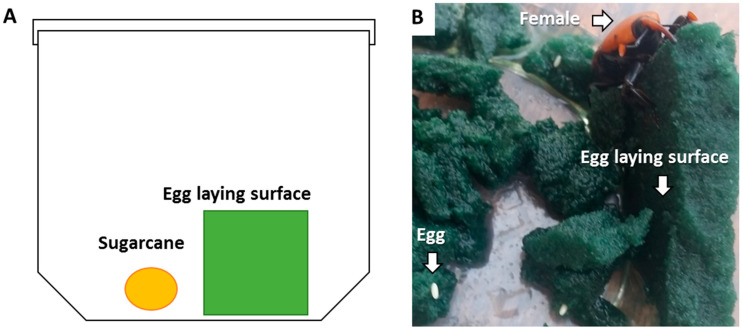
(**A**) Diagram of the experimental microcosm applied to evaluate the virulence of entomopathogenic fungi toward eggs and larvae under laboratory conditions (adapted from [23]). (**B**) Photograph of female weevil in the microcosm showing eggs laid in the foam piece.

This microcosm bioassay was developed in a previous study [23]. In brief, mated females were transferred individually to 500-mL plastic boxes with ventilated caps for six days. A piece of moist styrofoam (green styrofoam, 5 cm wide × 5 cm long × 1 cm high) and a piece of sugarcane (1 cm^3^, to serve as a food source) were put into each box (Figure 1A). For the control treatment, pieces of foam were soaked in a glass cup with 100 mL distilled water for 5 s. For the EPF-suspension treatments, pieces of foam were soaked in a glass cup with 100 mL suspension of 10^8^ conidia/mL for 5 s. For the EPF-rice granule treatments, pieces of foam were rubbed on all sides with 0.5 g of rice granules, an equivalent of 5 × 10^7^ conidia. Boxes were kept in an environmental chamber at 25 °C and 70% RH, with a 10 h:14 h dark/light regime for up to two weeks. Five days after the start of the experiment, females were removed from the boxes. Each female was placed in a clean box with sugarcane as food to monitor its mortality. Eggs were counted by examining the foam for oviposition holes made by the females. The pieces of foam were cut gently by hand, and eggs were counted for each replicate (Figure 1B). During the experimental period, egg hatching was monitored for up to 13 days, and the larvae that hatched in the foam were counted. Dead larvae or unhatched eggs were incubated in the dark at 25 °C in a 55 mm Petri dish lined with moist filter paper to confirm mycosis. There were 10 replicates per treatment and the experiment was conducted five times.

### 2.4. Greenhouse Experiment

**Figure 2:**
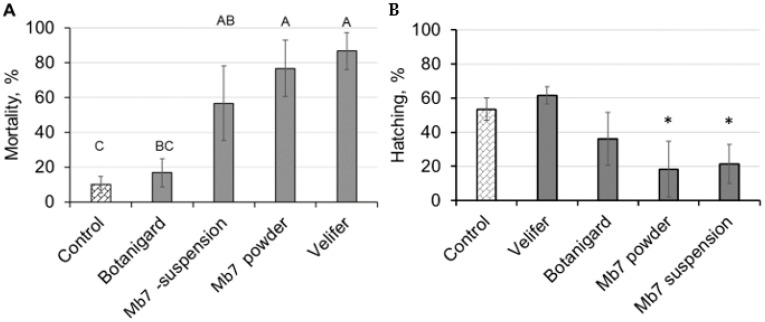
Effects of entomopathogenic fungal treatment of egg-laying surfaces on (**A**) mortality (average ± SE) of female RPW 1 week after inoculation and (**B**) egg-hatching rates (average ± SE) 10 days after inoculation. Different letters indicate significant difference, as determined by ANOVA followed by Tukey–Kramer HSD test. Asterisk denotes significant difference. Mortality: *F* = 11.6; DF = 4; *p* < 0.001. Hatching rate: *F* = 24.5; DF = 6; *p* < 0.001.

A total of 15 young *Washingtonia robusta* palms, 2 m in height, were placed in a climate-controlled greenhouse. Palms were watered daily, and dry leaves were pruned a week before the beginning of the experiment. At the start of the experiment, the palms were covered with 50-mesh nets (297 microns) to prevent the weevils’ movement between palms. The palms of the control group remained untreated. For dry conidial application, 100 g of sporulated rice granules were applied by hand to each palm to achieve complete coverage. For conidial suspension application, 500 mL of a 2.5 × 10^8^ conidia/mL suspension was sprayed manually to complete coverage of the palm. Mb7 was chosen for the greenhouse experiment since it exhibited the highest mortality rates of eggs (Figure 2) and is practicable for field application. The study was designed to segregate the formulation contribution and the Mb7 isolate contribution to the treatment efficacy. Two hours after application, six females and five males per palm were introduced into the nets. Weevils were monitored every two weeks. Dead weevils were removed, surface-sterilized, and incubated to assess rates of mycosis. New weevils were introduced instead of the dead ones to maintain the same number of total weevils throughout the experimental period. The palm infestation rate was assessed 60 days after treatment by dissection; the leaves were cut off with garden tools to reveal larvae and pupae. Temperature in the greenhouse was 28 ± 2 °C and RH ranged between 60 and 80%.

### 2.5. Data Analysis

All statistical analyses were performed using JMP^®^ Version 16 software (SAS Institute Inc., Cary, NC, USA). Results are presented as mean ± SE of replicate analyses and either represent or include at least three independent experiments.

For the laboratory experiment dataset, female mortality on day 7 post-inoculation and egg hatch on day 10 post-inoculation were analyzed. The square roots of the proportion data were arcsine-transformed and then subjected to analysis of variance (ANOVA) to examine the effects of the different treatments. If the effect was found to be significant (*p* < 0.05), the Tukey–Kramer HSD test was used for comparisons among means. 

For the greenhouse experimental dataset, adult mortality and the total number of larvae and pupae were analyzed by ANOVA, followed by the Tukey–Kramer HSD test for comparisons among means.

## 3. Results

### 3.1. Laboratory Experiments

The efficacy of the *B. bassiana*-based products and laboratory strain of *M. brunneum* (applied in a suspension or as sporulated rice) for the preventative control of RPW was evaluated using the experimental microcosm described in Section 2.3. Seven days after RPW, females were put in the oviposition boxes; three of the four EPF treatments had caused significant female mortality (Figure 2A; ANOVA: *p* < 0.0006, DF = 4, F = 11.6). Botanigard (*B. bassiana*-GHA) treatment resulted in female mortality similar to that observed in the control group. Ten days after females were put in the oviposition boxes, egg-hatching rates were only significantly lower for the Mb7 suspension and powder compared to the control (Figure 2B). The hatching rates for those treatments were 21% and 18%, respectively (Figure 2B; ANOVA: *p* < 0.0001, DF = 6, F = 24.54). Sporulation on eggs and larvae that hatched was observed in Botanigard and Mb7 treatments but with variations that did not allow statistical analysis. All non-hatched eggs in the Mb7 treatments exhibited signs of mycosis and sporulation.

### 3.2. Greenhouse Experiments

**Figure 3:**
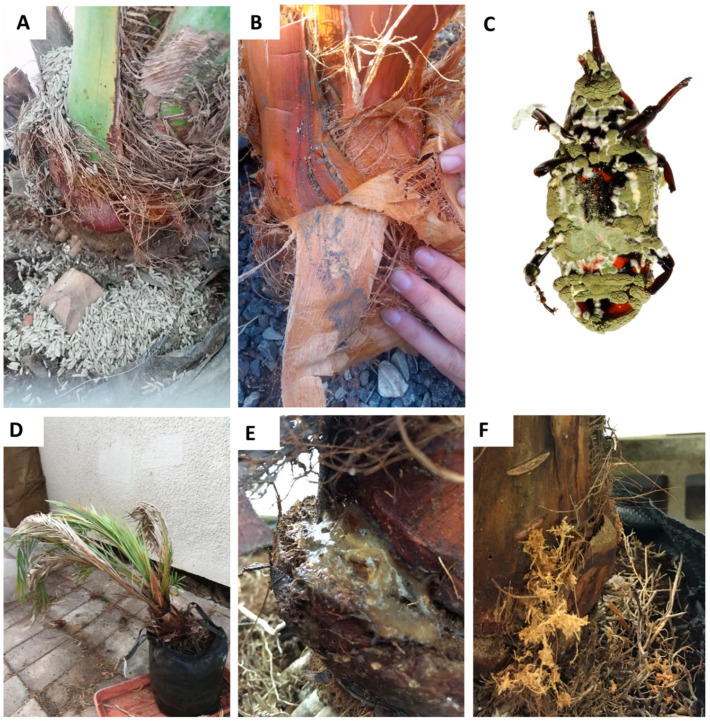
Experimental set-up for the greenhouse date palm experiment. (**A**) *Metarhizium brunneum* 7 (Mb7) sporulated-rice formulation applied to a date palm. (**B**) Dry powder of Mb7 conidia remaining between the layers of a date palm’s stem. (**C**) Adult RPW infested with *M. brunneum*. (**D**–**F**) Date palm from the control group showing signs of weevil infestation: (**D**) dry and fallen leaves; (**E**) typical secretions; (**F**) typical signs of boring.

The greenhouse experiment evaluated the virulence of Mb7 applied as either sporulated rice granules or conidial suspension; these were chosen because they exhibited the highest virulence toward eggs in the laboratory experiments (Figure 2). During the greenhouse experiment, conidia were visible on the fibrous threads and dried petioles (Figure 3A,B); dead weevils were collected, but the level of mortality was low, ranging from 6.25 to 10.5% (ANOVA: DF = 2, F = 0.29, *p* = 0.755; Table 2). Some adult cadavers were mycosed with *M. brunneum* (Figure 3C). Due to the scant mycosis that was exhibited by *M. brunneum*, we decided to dissect the palms 60 days into the experiment, when all palms in the control group exhibited clear signs of infestation: leaf collapse (Figure 3D), secretions (Figure 3E), and signs of boring (Figure 3F).

Secretion and boring signs were evaluated before dissection. All palms in the control group exhibited secretions, and 75% exhibited signs of boring (Table 2). There were no signs of boring in any of the Mb7-treated palms, and secretions were only observed in 25% of the palms treated with Mb7 suspension (Table 2). The control group had the highest average number of larvae per palm compared to both Mb7 treatments. However, the difference was only significant for the sporulated rice treatment (ANOVA: DF = 2, F = 5.43, *p* = 0.0283). The total number of pupae per palm was not significantly different between treatments (ANOVA: DF = 2, F = 0.648, *p* = 0.545; Table 2).

## 4. Discussion

Here, we report on utilizing the bioassay procedure to evaluate the efficacy of commercial products and lab preparations of EPF for RPW prevention by their transfer from the oviposition site to RPW eggs and larvae. In a previous study [18], adult mortality following inoculation with *M. anisopliae*-Ru reached 84.6–100% within 2 to 5 weeks of inoculation. That strain was not included in the current study as it was not available. Here, adult mortality was significant following exposure to Mb7 as conidia suspension or powder and Velifer^®^ (*B. bassiana*-PPRI 5339) but insignificant following exposure to Botanigard^®^ (*B. bassiana*-GHA). These differences in adult mortality can be attributed to the differences in the formulations of each product and the fungal strains examined. Formulation adjuvants are well recognized for contributing to pest mortality [25,26,27]. As the exact adjuvant composition of the two commercial products was not disclosed, we cannot discuss it specifically.

Current knowledge of entomopathogenic fungal characteristics contributing to its virulence and host range is limited. In previous studies, we found that non-virulent *Metarhizium* species are unable to develop beyond pre-penetration events: germination and appressorium formation. Therefore, on a resistant tick host, Mb7 was able to germinate and produce appressoria, but it failed to penetrate the cuticle [28]. Similarly, *M. majus* was able to germinate on the cuticle but then died without penetrating the cuticle. As the RPW eggs are physiologically identical, we suggest that the observed variation in its infection relates to pre-penetration events in the fungi. A potential alteration in the adhesion rate between the examined EPF, and the fungal ability to breach the egg shell barrier by producing degrading enzymes that are key factors for broad host range [29,30]. We are aware of the intra species variations between Mb7 and MbK in terms of disease progression and pathogenicity; however, these variations cannot be solely explained by genomic variations [31]. Also, the observed variation between the two *B. bassiana* isolates evaluated in this study could also be unexplainable by genomic variations. Overall, we hypothesize that other drivers exist for fast adaptation and responses in the fungal cell such as epigenetic regulation and dormancy break but these aspects are being evaluated under different frameworks [29,31].

Based on the conclusions from the lab bioassay and the previous study which indicated higher virulence of *M. brunneum* over *B. bassiana* to RPW [18], with more evidence of its ovicidal performances in different arthropods [32,33], we examined the potential of *M. brunneum* as a preventative treatment for RPW infestation under greenhouse conditions. The palm trees were infested with adult RPW as described by Dembilio et al. [22]. All of the palm trees from the control group showed signs of infestation—secretions and wilted dry leaves. Sporulated rice was superior to a conidial-suspension spray for preventing infestation by RPW. Although we were not able to examine the mechanical transmission of conidia toward eggs and larvae, we suggest that the positive results observed in the greenhouse experiment were due to the transfer of conidia from the treated surface of the palm to the oviposition site when the females laid their eggs. The acquisition of conidia by individual weevils, as described in [22,23], could not be confirmed in our greenhouse experiment because not all of the released adult RPW could be recovered, mortality rates were low, and some adult cadavers were mycosed probably with the applied fungus. High efficacy of the sporulated-rice formulation has been observed in field trials involving RPW [21,34] and the black palm weevil *Rhynchophorus bilineatus* [35], indicating the potential of this method for reducing other palm weevil species but also a potential for other palm pests such as lepidopteran and scarabs.

The bioassay developed in this study was used to test the effects of different strains and commercial formulations of EPF on female RPW and their progeny and to assess the commercial potential of the preventative use of EPF. The results were in accordance with those of Hajjar et al. [36], who reported a median lethal time (LT50) of 4.15 days for the *B. bassiana* product referred to as Velifer^®^ in this work for adult RPW at a concentration of 10^8^ conidia/mL. In our study, that same concentration of Velifer^®^ resulted in >80% female mortality seven days after inoculation. Female mortality depended on the product used but probably not on the formulation; Mb7, as a suspension or powder, caused similar mortality rates of females and similar egg-hatching rates. Of the three strains and four formulations examined, only the *M. brunneum* laboratory preparations significantly reduced egg hatching. This finding is in accordance with Gindin et al. [18], who found that *M. anisopliae* strains are more virulent toward larvae than *B. bassiana* strains. However, the differences between the various products, in terms of their formulations, may influence the horizontal transfer of conidia and, consequently, the observed efficacy of the different treatments [27]. 

The results of the current study encourage further research to develop EPF applications as a preventative measure for the control of RPW on palm trees. In future study, the preventative efficacy of both *M. brunneum* and *B. bassiana-based* products should be evaluated along with disseminating the knowledge to date farmers. Questions for further investigations include: How long can conidia persist under field conditions? Would EPF treatments be more cost-effective if they were focused on oviposition sites? Is the strategy effective on other palm pests, such as the family Scarabaeidae and the order Lepidoptera? Even in a situation in which EPF would account for a significant share of the total pesticide applied to palm trees, other issues, such as early detection of infestations and reactive treatments for infested palms, still need to be explored.

## 5. Conclusions

Examination of the efficacy of the proposed preventative strategy on palms on which adult RPW were released revealed evident inhibition of palm infestation by RPW larvae. The efficacy of the different EPF products for reduced egg hatching and female mortality varied widely. Of the examined products, a dry formulation of *M. brunneum* is the most promising candidate for field evaluation of RPW prevention, but such a product is not available in the Israeli market. Overall, our results suggest that EPF-based products can serve as a valuable and safe preventative means that can be incorporated into a broader management strategy toward RPW.

## Figures and Tables

**Table 1:** Examined entomopathogenic fungal products and strains: biological and technical details.

Species	Strain	Country of Origin	Source	Trade Name	Formulation
*Beauveria bassiana*	PPRI 5339	South Africa	BASF	Velifer	Emulsified concentrate
*Beauveria bassiana*	GHA	USA	LAM International Corporation	Botanigard	Emulsified concentrate
*Metarhizium brunneum*	Mb7 *	Israel	Sporulated rice produced in the laboratory	None	(1) Dry conidia granules (2) Conidial suspension

* Tested in [18,23].

**Table 2:** Prevalence of signs of infestation on plants in the greenhouse experiment (secretion and boring) and mean (±SE) of cumulative mortality of adults during the experiment, and total larvae and total pupae found on those palms 60 days after application *.

Treatment	Secretion (%)	Signs of Boring (%)	Cumulative Mortality of Adults	Total Larvae per Palm	Total Pupae per Palm
Control	100	75	6.25 ± 2.5 a	8.5 ± 3.3 a	1.5 ± 1.5 a
Conidial suspension	25	0	10.5 ± 2.9 a	1 ± 1.9 ab	3 ± 1.8 a
Sporulated rice	0	0	10 ± 6.4 a	0 b	0 a

* For each life stage, values followed by a different letter are significantly different between treatments as determined by ANOVA followed by Tukey–Kramer HSD test. Adults: DF = 2, F = 0.29, *p* = 0.755. Larvae: DF = 2, F = 5.43, *p* = 0.0283. Pupae: DF = 2, F = 0.648, *p* = 0.545.

## Data Availability

Correspondence and requests for materials should be addressed to D.M.
